# The Human Energy Balance: Uncovering the Hidden Variables of Obesity

**DOI:** 10.3390/diseases13020055

**Published:** 2025-02-13

**Authors:** Nikolaos Theodorakis, Maria Nikolaou

**Affiliations:** 1 NT-CardioMetabolics, Clinic for Metabolism and Athletic Performance, 47 Tirteou Str., 17564 Palaio Faliro, Greece; 2 Department of Cardiology & Preventive Cardiology Outpatient Clinic, Amalia Fleming General Hospital, 14, 25th Martiou Str., 15127 Melissia, Greece; m.nikolaou@flemig-hospital.gr; 3 School of Medicine, National and Kapodistrian University of Athens, 75 Mikras Asias, 11527 Athens, Greece

**Keywords:** diet-induced thermogenesis, energy balance, metabolism, non-exercise activity thermogenesis, obesity, precision nutrition, spendthrifty, thermic effect of food, thrifty, weight loss

## Abstract

Obesity has emerged as a global epidemic, creating an increased burden of weight-related diseases and straining healthcare systems worldwide. While the fundamental principle of energy balance—caloric intake versus expenditure—remains central to weight regulation, real-world outcomes often deviate from simplistic predictions due to a multitude of physiological and environmental factors. Genetic predispositions, variations in basal metabolic rates, adaptive thermogenesis, physical activity, and nutrient losses via fecal and urinary excretion contribute to interindividual differences in energy homeostasis. Additionally, factors such as meal timing, macronutrient composition, gut microbiota dynamics, and diet-induced thermogenesis (DIT) further modulate energy utilization and metabolic efficiency. This Perspective explores key physiological determinants of the energy balance, while also highlighting the clinical significance of thrifty versus spendthrifty metabolic phenotypes. Key strategies for individualized weight management include precision calorimetry, circadian-aligned meal timing, the use of protein- and whole food diets to enhance DIT, and increases in non-exercise activity, as well as mild cold exposure and the use of thermogenic agents (e.g., capsaicin-like compounds) to stimulate brown adipose tissue activity. A comprehensive, personalized approach to obesity management that moves beyond restrictive caloric models is essential to achieving sustainable weight control and improving long-term metabolic health. Integrating these multifactorial insights into clinical practice will enhance obesity treatment strategies, fostering more effective and enduring interventions.

## 1. Introduction

Obesity has emerged as a global public health crisis, affecting individuals across all demographics and socioeconomic strata. Its prevalence has escalated in tandem with rapid urbanization, altered dietary patterns, and increasingly sedentary lifestyles, leading to a dramatic rise in weight-related chronic conditions such as type 2 diabetes, cardiovascular disease, and certain cancers. Governments and health organizations worldwide recognize that obesity represents not just a personal health concern but also a pressing societal and economic burden, straining healthcare systems and driving up costs. Despite heightened awareness and numerous public health campaigns, obesity rates continue to surge, highlighting the need for innovative, evidence-based strategies to address the complexities underlying this pandemic [1].

Efforts to manage body weight often rely on the simplistic notion of balancing calories in versus calories out, yet real-world outcomes frequently deviate from these predictive formulas. Evidence illustrates that energy intake is neither fully accounted for by standard food labels nor is all of the recorded energy entirely absorbed, while energy expenditure comprises multiple overlapping processes, some of which remain unmeasured in everyday practice [1,2]. Specifically, adaptive metabolic mechanisms, ranging from diet-induced thermogenesis (DIT)/thermic effect of food (TEF) to adaptive thermogenesis (AT) and Luxuskonsumption represent further attempts to predict weight outcomes [1,3]. Moreover, a significant fraction of ingested nutrients may be excreted in feces and urine, reducing the net “absorbed” energy available for metabolism and storage [2].

This manuscript offers a novel perspective on the major determinants that affect human energy balance and subsequently weight loss. By integrating these emerging insights into a unified framework, it underscores the need for holistic, individualized strategies to account for the multifaceted nature of human metabolism. In doing so, the paper aims to inform more effective clinical interventions and stimulate further research into underexplored contributors to obesity risk and resilience.

## 2. Established Drivers of Obesity

Some of the most well-established factors leading to obesity include excessive energy intake, reduced physical activity, hormonal dysregulation, genetic predisposition, and gut microbiota alterations. While obesity is a multifactorial condition influenced by both lifestyle and biological determinants, these key contributors interact to create an environment that promotes energy surplus and weight gain. 

The rise in the prevalence of obesity is strongly linked to increased energy intake, driven by the widespread availability of calorie-dense, highly palatable foods. Modern dietary patterns are characterized by an excessive consumption of ultra-processed foods rich in refined carbohydrates, unhealthy fats, and added sugars, which contribute to hyperphagia and a positive energy balance. Moreover, the increased portion sizes and high glycemic loads within many modern diets can promote insulin secretion, favoring fat storage while suppressing postprandial energy expenditure. Additionally, the rewarding properties of certain foods, mediated by dopaminergic signaling in the brain, can lead to compulsive overeating and difficulty in maintaining dietary restraint. The combination of these factors fosters a persistent caloric surplus, ultimately leading to weight gain and obesity [4].

Concurrently, a significant reduction in physical activity has contributed to the obesity epidemic. Urbanization, technological advancements, and changes in occupational environments have led to an overall decline in energy expenditure through decreased engagement in occupational, recreational, and household activities. The increasing reliance on motorized transport, prolonged screen time, and sedentary behaviors have further exacerbated the energy imbalance, as non-exercise activity thermogenesis (NEAT) has markedly declined in many populations. The shift from physically demanding jobs to more sedentary professions, coupled with the decreased need for manual labor in daily activities, has resulted in lower total daily energy expenditures [5].

Obesity is also influenced by dysregulation of key hormones involved in appetite regulation, energy balance, and fat metabolism. Leptin, an adipocyte-derived hormone, plays a crucial role in signaling satiety and regulating energy expenditure; however, in obesity, leptin resistance impairs these feedback mechanisms, leading to persistent hunger and a reduced metabolic rate. Similarly, ghrelin, a stomach-derived hormone that stimulates hunger, is often dysregulated in individuals with obesity, contributing to increased food intake. Insulin resistance, commonly observed in obesity, exacerbates metabolic dysfunction by promoting lipogenesis and impairing glucose homeostasis. Additionally, stress-related elevations in cortisol levels can drive central adiposity through increased appetites and alterations in substrate metabolism. Collectively, these hormonal imbalances create a metabolic environment that favors weight gain and challenges sustained weight loss efforts [6,7,8].

Beyond lifestyle and hormonal factors, genetic predispositions play a crucial role in determining an individual’s susceptibility to obesity. Genome-wide association studies (GWASs) have identified numerous obesity-related genetic variants, including those in the FTO and MC4R genes, which are associated with an increased appetite, reduced satiety, and alterations in energy expenditure. Additionally, epigenetic modifications, influenced by early-life nutrition and environmental exposures, can alter gene expression patterns related to adipogenesis and metabolic regulation. Recent research has also highlighted the gut microbiota as a key player in obesity pathophysiology. An imbalance in gut microbial composition, characterized by a higher ratio of Firmicutes to Bacteroidetes, has been linked to increased energy harvest from the diet, chronic low-grade inflammation, and altered lipid metabolism. These genetic, epigenetic, and microbiome-related factors interact with environmental influences, further complicating obesity’s multifactorial nature [9,10].

## 3. Determinants of the Human Energy Balance

The dominant energy balance model holds that a positive energy balance (calories in > calories out) drives weight gain, while a negative balance drives weight loss [11]. In clinical practice, however, weight change often deviates from predictions based purely on declared caloric intake and resting energy expenditure [3]. These discrepancies arise partly from unaccounted processes:

▪ Basal metabolic rate (BMR) fluctuations beyond predictive equations;
▪ DIT/TEF;
▪ Adaptive thermogenesis (AT) and Luxuskonsumption;
▪ Brown Adipose Tissue (BAT) thermogenic activity;
▪ Physical activity-related energy expenditure (PEE): NEAT and exercise activity thermogenesis (EAT);
▪ Fecal and urinary energy losses.

**Figure 1:**
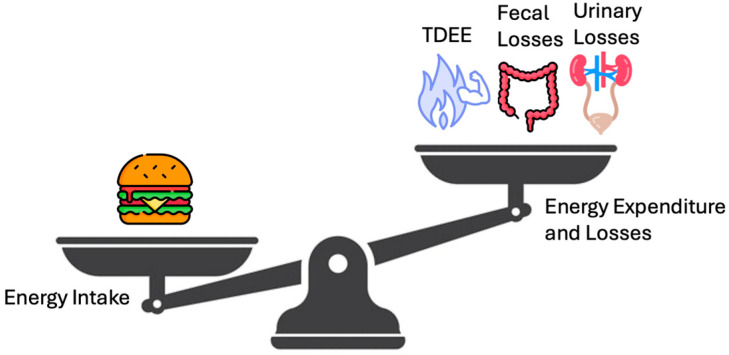
Human Energy Balance: Intake vs. expenditure and losses. A simplified scale diagram comparing energy intake (e.g., symbolized by a food icon) to total daily energy expenditure (TDEE) plus excreted losses. The balance (or imbalance) determines net energy gain or loss.

**Figure 2:**
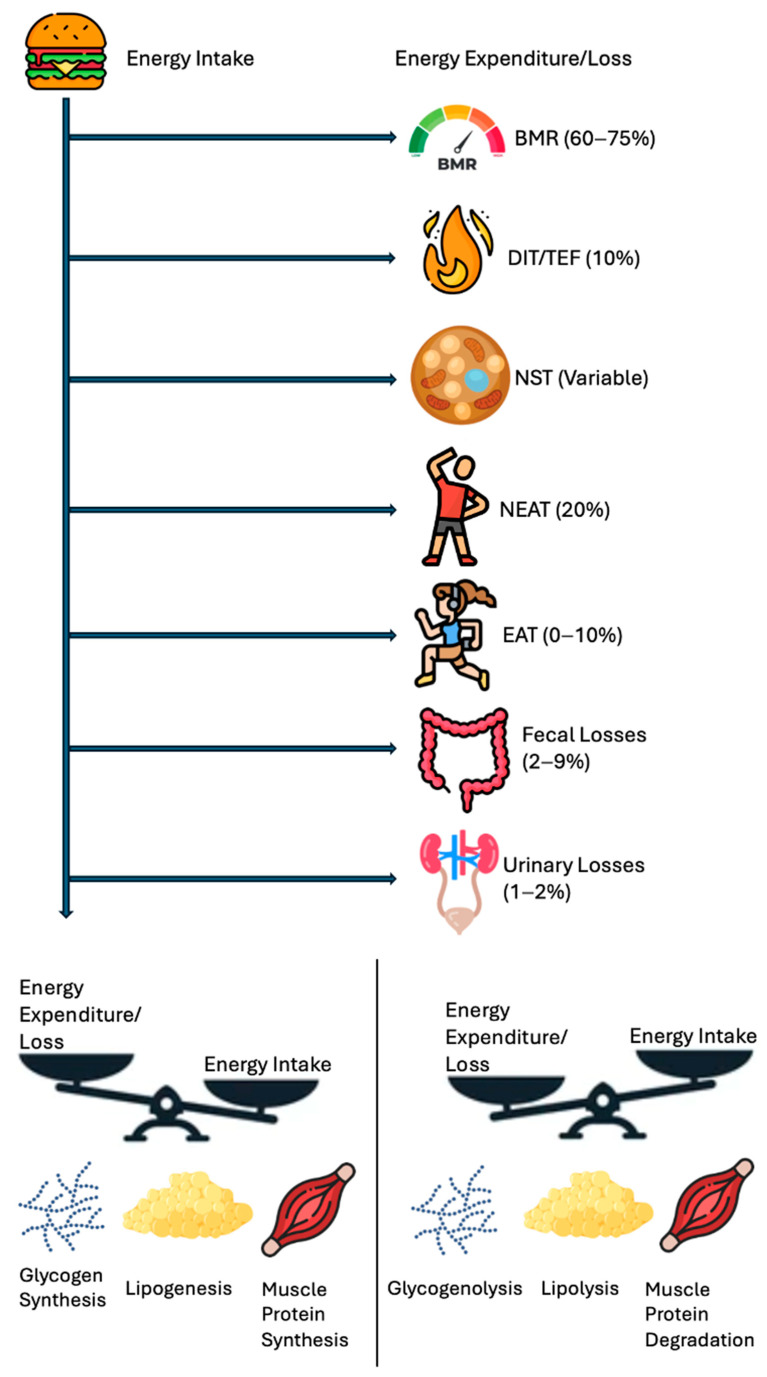
Human energy balance: pathways of expenditure, storage, and utilization.

Figure 1 highlights the energy balance in a simpler manner, a scale tipping in favor of intake vs. expenditure plus loss, underscoring that net calorie availability is the ultimate determinant of weight change. Figure 2 depicts the vertical breakdown of ingested calories: one portion is lost via feces and urine, another supports the BMR, and another fraction is channeled into EAT and NEAT, while BAT activity represents thermogenesis due to the uncoupling of oxidative phosphorylation.

A schematic breakdown of the human energy balance, showing how ingested calories are partitioned into BMR, DIT/TEF, NST, NEAT, EAT, and losses (fecal, urinary). S positive caloric remainder leads to increased fat/muscle storage, while a negative remainder draws on glycogen and fat stores. Abbreviations: basal metabolic rate, BMR; diet-induced thermogenesis, DIT; exercise activity thermogenesis, EAT; non-exercise activity thermogenesis, NEAT; non-shivering thermogenesis, NST; thermic effect of food, TEF.

### 3.1. BMR

The BMR is generally recognized as the single largest contributor to total daily energy expenditure in sedentary individuals, often accounting for roughly 60–75% of total caloric output. Despite the prevalence of standardized prediction equations for estimating the BMR, actual values can differ substantially among individuals due to a variety of influences [12]. Genetic factors, such as polymorphisms in thyroid hormone signaling pathways, may modulate the rate at which cells consume oxygen and produce energy, thereby altering baseline caloric needs. Differences in body composition are also pivotal; individuals with higher proportions of lean mass tend to exhibit an elevated BMR because muscle tissue has a greater metabolic demand than adipose tissue. Additionally, health status—ranging from metabolic disorders to subclinical inflammation—can either increase or suppress basal metabolism, sometimes leading to surprising discrepancies when using standard predictive formulas [13].

Such deviations can undermine the effectiveness of a diet plan if the true baseline energy requirement is inaccurately assumed to be higher or lower than it actually is [1,14]. For this reason, direct measurements of the resting metabolic rate (RMR) via indirect calorimetry can be invaluable, particularly for individuals facing challenges in weight management. By measuring oxygen consumption and carbon dioxide production, indirect calorimetry yields a precise snapshot of how many calories are needed to maintain critical physiological functions. Moreover, this measurement sheds light on the proportions of energy derived from carbohydrates, fats, and proteins—information that can be harnessed to tailor macronutrient ratios and caloric prescriptions to a patient’s unique metabolic profile. Ultimately, the nuanced understanding of BMR and substrate utilization provided by indirect calorimetry helps clinicians to optimize dietary interventions and improve the likelihood of sustainable weight control [1,14].

Meal timing can significantly influence the BMR and energy expenditure. A randomized crossover trial in adults with overweight/obesity found that late eating increased hunger, reduced 24 h leptin levels, and elevated the ghrelin–leptin ratio, suggesting a stronger drive for food intake. Additionally, late eating led to a measurable decline in total energy expenditure and core body temperature, with no compensatory increases during sleep, indicating an overall reduction in the metabolic rate. Late eating also altered adipose tissue gene expression, downregulating lipolysis-related genes (PLD6, DECR1, ASAH1, ABHD5), and upregulating adipogenesis-related genes (GPAM, ACLY, AACS, CERK), along with changes in p38, Mitogen-Activated Protein Kinase (MAPK), Transforming Growth Factor Beta (TGF-β), autophagy, and tyrosine kinase signaling, favoring fat storage. These findings highlight that meal timing, independent of caloric intake, plays a critical role in metabolic regulation and obesity risk, supporting the importance of aligning food intake with circadian rhythms [15].

### 3.2. DIT/TEF

DIT, also referred to as TEF, is a postprandial rise in the metabolic rate due to the energy required for digestion, absorption, transport, metabolism, and the storage of nutrients. While DIT contributes on average ~10% of the daily total energy expenditure, its magnitude can vary significantly based on macronutrient composition, meal size, food processing, and individual metabolic factors [3].

#### 3.2.1. Protein

▪ TEF range: ~20–30% (up to 35%) of the ingested protein’s caloric content [3].
▪ Protein digestion involves proteolytic enzymes in the stomach and small intestine, followed by the active transport of amino acids which requires adenosine trisphosphate (ATP) [16].
▪ Deamination of amino acids in the liver (removal of the amino group) and subsequent urea formation require ATP, which increases the postprandial metabolic rate [17].
▪ The insulin response to protein intake drives nutrient uptake and can mildly elevate energy expenditure during and shortly after a meal. Protein also stimulates a robust glucagon response, modulating postprandial thermogenesis [18].
▪ Certain amino acids, especially leucine, can directly modulate the mammalian target of rapamycin and muscle protein synthesis, further elevating postprandial energy demands [19].

#### 3.2.2. Carbohydrates

▪ TEF range: ~5–10% (up to ~15%) of ingested carbohydrate calories [3].
▪ Carbohydrate digestion begins with salivary amylase and continues in the small intestine.
▪ The absorption of monosaccharides (e.g., glucose) occurs via active transport or facilitated diffusion, which require modest energy expenditure [20].
▪ Further metabolism of glucose—storage as glycogen or partial conversion to fat (de novo lipogenesis)—demands additional ATP [21].
▪ The insulin response to carbohydrate intake drives nutrient uptake and can mildly elevate energy expenditure during and shortly after a meal [21].
▪ The glycemic index (GI) influences carbohydrate-induced thermogenesis by modulating the speed at which glucose enters the bloodstream and triggers insulin release [1].
▪ Lower-GI foods (e.g., minimally processed grains, legumes) tend to provoke a slower, more sustained postprandial insulin response, which may extend or modestly increase the TEF compared to high-GI foods, whose rapid absorption often leads to a sharp insulin spike but a less prolonged TEF [1,3].

#### 3.2.3. Lipids

▪ TEF range: typically ~0–3% (up to 5%) of ingested fat calories [3].
▪ Fat digestion, chiefly via pancreatic lipase and bile salt emulsification, though essential, is relatively efficient and less energetically costly compared to protein and carbohydrate processing [22].
▪ Absorbed fatty acids and monoglycerides are packaged into chylomicrons, which requires some energy investment but is considerably lower than the processes required for protein turnover or carbohydrate metabolism [22].
▪ Once in circulation, the storage of fatty acids in adipose tissue (via lipoprotein lipase-mediated uptake) is also efficient, resulting in a comparatively low thermic effect [22].

### 3.3. Additional Factors Affecting TEF

There are additional multifactorial influences on DIT and postprandial energy expenditure, underscoring the importance of meal composition, physical activity, age, and circadian rhythm in metabolic regulation (Table 1). Older adults exhibit significantly a lower TEF and BMR compared to younger individuals, contributing to reduced daily energy expenditure and potential weight gain risks [23]. Habitual physical activity is associated with a higher TEF, reinforcing its role in enhancing postprandial thermogenesis and weight management [24]. Meal composition and structure play a crucial role, with medium-chain triglycerides (MCTs) producing greater thermogenic effects than long-chain triglycerides (LCTs) and whole food meals enhancing the TEF more than processed foods [25,26]. Additionally, larger single-bolus meals elicit greater DIT responses compared to smaller, more frequent meals, suggesting potential metabolic advantages from meal timing strategies. Circadian rhythms also impact TEF, with morning meals generating a higher TEF than evening meals, though this effect may be largely attributed to underlying metabolic fluctuations rather than meal timing alone [27,28]. These findings emphasize the necessity of integrating meal composition, timing, and physical activity into personalized dietary strategies to optimize metabolic efficiency and support long-term weight management.

### 3.4. BAT Thermogenesis

BAT is recognized as a key thermogenic site for non-shivering thermogenesis, helping dissipate excess energy and potentially contributing to weight regulation when a person is sufficiently active. Although the concept of human BAT-mediated DIT remains controversial, recent work suggests that a postprandial rise in BAT oxidative metabolism can parallel its cold-induced response. Mechanistically, BAT is primarily controlled via sympathetic outflows and β-adrenergic signaling, with norepinephrine acting on abundant mitochondria that express uncoupling protein-1 (UCP1) to generate heat rather than ATP [29].

Nutritional agents capable of activating BAT have garnered particular attention, especially those that appear to amplify sympathetic activity. In humans, compounds such as grains of paradise, capsaicin, and capsinoids can raise whole-body energy expenditure through BAT activation in individuals displaying readily detectable BAT on 18F-FDG PET imaging. Additional studies have shown that the ingestion of capsaicin-like molecules consistently promotes thermogenesis, partially via transient receptor potential channels, and appears more effective when functional BAT is present. Such findings align with broader evidence indicating that BAT recruitment, whether through cold exposure or select dietary factors, holds promise for modestly enhancing daily caloric output [30,31].

Moreover, repeated cold acclimation in humans can elevate BAT mass or activity, as supported by multi-week protocols that heighten cold-induced thermogenesis. Similar phenomena may arise from the regular consumption of spicy food compounds, exemplified by capsinoids, which have been associated with improved non-shivering thermogenesis over time. Recent techniques also suggest a link between BAT activation and muscle metabolic profiles, highlighting that the synchronized stimulation of both tissues may offer metabolic advantages. Although its exact energetic impact remains smaller than once hoped, BAT nevertheless adds an extra layer of metabolic flexibility to daily energy turnover [30,31].

Practical applications include combining mild cold exposure, targeted dietary factors, and conventional lifestyle interventions to tap into BAT’s thermogenic capacity. Nonetheless, the high variability in BAT prevalence, mass, and responsiveness makes standardized approaches challenging. Additionally, exclusive reliance on BAT-induced energy dissipation is unlikely to counteract a significant caloric excess. From a clinical standpoint, BAT-supportive strategies should be regarded as complementary tools in a multifaceted approach to obesity and metabolic syndrome, rather than as stand-alone solutions [29,30,31].

### 3.5. NEAT

PEE includes NEAT and EAT. NEAT encompasses the range of energy expenditure that arises from activities of daily living that fall outside of structured exercise and basal metabolism, accounting for roughly 20% of total caloric output. NEAT has emerged as a crucial yet often underestimated element of daily energy expenditure, particularly in individuals whose EAT remains low or negligible. By definition, NEAT captures the energy expended above the RMR and DIT, encompassing a wide spectrum of spontaneous, unstructured, and relatively low-intensity movements such as standing, fidgeting, household chores, and general ambulation. Although each of these movements might appear trivial when viewed in isolation, they can cumulatively account for meaningful variations in total daily energy expenditure between and within individuals [32]. 

When people spend extended periods in physically inactive postures—such as prolonged sitting—NEAT naturally decreases, contributing to a sustained positive energy balance. Studies in both lean and obese populations consistently show that individuals with obesity tend to have lower baseline NEAT, in part because of an increased inclination toward sedentary behaviors. Importantly, NEAT may fluctuate based on a host of personal and environmental factors, including occupation (e.g., deskbound vs. physically demanding jobs), cultural norms around movement, and built environment features that either facilitate or discourage incidental activity. Such diversity in lifestyle contexts underpins why NEAT levels differ substantially from person to person [32,33].

Given the recognized challenges of sustaining structured exercise regimens, recent attention has turned to incorporating NEAT-enhancing strategies for weight management and cardiometabolic health. Encouraging regular breaks from sitting, adopting active workstations, and performing simple tasks—such as light walking or pacing—during daily routines can collectively elevate energy expenditure with minimal disruption. Although NEAT generally involves lower intensities than traditional exercise training, frequent episodes of these small, repeated movements accumulate over the course of the day. Consequently, addressing inactivity from the standpoint of increasing NEAT may offer an alternative avenue for individuals who find it difficult to adhere to moderate-to-vigorous exercise guidelines [32,33,34].

Quantifying NEAT precisely remains challenging, as many assessment methods were initially geared to measure more structured physical activities rather than the micro-level movements that define NEAT. Nonetheless, advanced tools like accelerometers, multi-sensor armbands, and metabolic chambers are increasingly used to parse out the minute fluctuations in posture and movement central to NEAT. However, in daily clinical or field settings, questionnaires remain the most commonly employed approach due to their low cost, feasibility, and straightforward administration, enabling rapid data collection across large populations. Several instruments, including the International Physical Activity Questionnaire (IPAQ) and the Global Physical Activity Questionnaire (GPAQ), capture self-reported behaviors that can be mapped onto NEAT-related activities, though they inherently rely on participant recall and may underestimate micro-level fidgeting or posture changes [32,33,34,35,36]. In tandem with these surveys, researchers sometimes apply physical activity ratios (PARs)—standardized multipliers of the RMR—to estimate the energy cost of specific tasks. For instance, sitting quietly might have a PAR close to 1.0, meaning it approximates basal energy demands, whereas light walking or mild household chores might range from 2.0 to 4.0 times the RMR, depending on the intensity of movement. By multiplying the time reported participating in a particular activity by its designated PAR and by an individual’s known or predicted RMR, one can approximate the thermic contribution of those low-grade movements more precisely. Although PAR-based calculations can yield an improved understanding of how different daily tasks contribute to NEAT, they remain subject to the same self-report limitations that characterize questionnaire-based approaches. Future research will need to refine these measurement strategies—questionnaires, PAR assignments, and device-based monitoring—to better capture the diversity of NEAT behaviors and evaluate whether targeted NEAT interventions translate into meaningful improvements in weight control and metabolic parameters. Over time, such efforts could clarify how best to integrate NEAT within broader lifestyle interventions aimed at reducing the prevalence of obesity and mitigating its associated health risks [32,33,34,35,36].

### 3.6. EAT

EAT refers to the energy spent during structured physical activities, ranging from moderate-intensity continuous exercise (MICE) to high-intensity interval exercise (HIIE) and sprint interval exercise (SIE), as well as resistance training. Although exercise typically accounts for 0–10% of the total daily energy output, these activities can acutely raise overall energy expenditure—sometimes substantially—yet compensatory behaviors may diminish the net benefits. Individuals might unintentionally reduce non-exercise activity after intense workouts, or increase caloric intake in response to heightened appetite. Consequently, exercise regimens designed to monitor and mitigate these compensations often produce more consistent outcomes for weight management [2,37].

Beyond the immediate calories burned, exercise can also affect energy metabolism through excess post-exercise oxygen consumption (EPOC). A recent systematic review of 22 studies examined the effect of exercise intensity—HIIE vs. MICE vs. SIE—on EPOC, splitting investigations into those evaluating short-duration EPOC (≤3 h) and long-duration EPOC (>3 h). Among short-duration evaluations that subtracted baseline energy expenditure (EE), HIIE produced ~136 kJ of post-exercise EE, while MICE averaged ~101 kJ. SIE reached ~241 kJ, compared with ~151 kJ for MICE. In long-duration measurements, HIIE resulted in ~289 kJ, whereas MICE averaged ~159 kJ; no long-duration data were available for SIE vs. MICE comparisons. These findings suggest that EE from EPOC tends to be greater following HIIE and SIE than with MICE, and that longer measurement intervals may reveal higher EPOC totals. More standardized methodologies remain necessary to delineate the effective duration of EPOC after such training protocols [38,39].

In one investigation, participants who engaged in 80 min of endurance exercise at approximately 70% of their maximal oxygen uptake exhibited sustained elevations in oxygen uptake for up to 12 h, with potential metabolic effects persisting for as long as 24 h. Notably, post-meal oxygen consumption was also elevated following exercise, indicating that endurance training may enhance metabolic efficiency even during subsequent nutrient intake. These findings reinforce the importance of both exercise intensity and duration in modulating post-exercise metabolic responses, suggesting that structured training strategies can contribute to an improved energy balance and long-term metabolic health [40].

### 3.7. Fecal and Urinary Energy Losses

Fecal and urinary energy losses represent an often-underrecognized dimension of the human energy balance. While caloric intake is usually treated as a total figure drawn from food labels or nutrient databases, a fraction of these ingested calories may never be absorbed. In controlled inpatient feeding trials, fecal energy loss in healthy adults has ranged from approximately 2% to 9% of total consumed calories, translating to anywhere between 80 kcal/day and 500 kcal/day among individuals in an overfeeding scenario. Such a gap can be pivotal in explaining inter-individual variability in weight gain because those with higher fecal losses effectively divert a larger share of their caloric intake away from storage pathways. Similar, though generally smaller, differences are observed in urinary energy losses, which on average comprise about 1% to 2% of ingested calories. Elevated urea excretion—often linked to high-protein dietary patterns—can slightly reduce net energy retention by accelerating the disposal of nitrogen-containing compounds. While these differences may appear modest at first glance, they accumulate over time and can substantially alter the expected outcome of any given nutritional regimen. This phenomenon accounts for why some individuals gain significantly less weight than predicted under caloric surplus conditions, revealing that not all consumed energy is inevitably destined for metabolic utilization or storage [2].

## 4. Thrifty vs. Spendthrifty Phenotypes, Luxuskonsumption, and AT

The notion of “thrifty” and “spendthrifty” phenotypes has gained renewed attention to explain how individuals respond differently to a caloric surplus or deficit. Thrifty phenotypes tend to conserve energy through reduced thermogenesis and efficient nutrient absorption, thereby favoring weight gain and making weight loss more challenging. Spendthrifty phenotypes, on the other hand, display higher levels of energy dissipation—whether through increased fecal losses, amplified TEF, or heightened BAT activity—leading to relative resistance to weight gain and more rapid weight loss. Although the precise biological mechanisms remain under investigation, variations in sympathetic nervous system tone, thyroid hormone sensitivity, and intestinal nutrient handling likely contribute [2,41].

Also relevant to this spectrum is the concept of constitutional thinness, characterized by a persistently low body mass index in the absence of apparent eating disorders or malabsorption syndromes. Individuals with constitutional thinness typically display normal or even robust appetites but fail to accumulate significant adipose stores, suggesting an enhanced capacity for energy dissipation or excretion. Although underlying factors may include genetic predispositions and subtle differences in nutrient absorption or hormone regulation, constitutional thinness can be viewed as a practical example of a spendthrifty-like phenotype, wherein weight gain proves to be notably difficult despite substantial caloric intakes [42].

Luxuskonsumption refers to an adaptive surge in total energy expenditure during periods of chronic overfeeding—an effect that surpasses the energy cost of simply carrying a larger body mass. While historically it was believed that some individuals could dramatically “burn off” excess calories through heightened thermogenesis, recent data suggest that significant overfeeding-induced metabolic boosts are relatively rare [1]. In one controlled overfeeding study (8 weeks of overfeeding at 40% above baseline energy needs), the mean increase in 24 h energy expenditure was only about 23 kcal/day above baseline, implying that few people truly harness a major luxury consumption mechanism capable of completely neutralizing a caloric surplus [43]. Nevertheless, certain individuals do exhibit a measurable thermogenic response when they are chronically overfed, and these higher responders gain less fat than predicted. This adaptive variability highlights why conventional calorie-based predictions often fail to account for real-world outcomes and underscores the importance of individualized assessments in weight management [1].

AT can pose a formidable challenge in sustaining weight loss by suppressing the basal metabolic rate beyond what is attributable to the decline in lean mass [1]. Chronic caloric restriction, for instance, lowers leptin and triiodothyronine levels and dampens sympathetic outflow, while amplifying the orexigenic signal ghrelin—all of which collectively drive individuals toward weight regain despite continuing dietary and exercise regimens. Although these adaptive responses often lead to weight-loss plateaus or even reversals of progress, a recent systematic review of thirty-three studies (n = 2528) suggests that the magnitude and clinical significance of AT may vary widely [44]. Although AT was identified in most of the included trials, the more rigorously designed investigations tended to report modest or statistically non-significant effects. Furthermore, the review indicates that AT may wane or disappear altogether after periods of weight stabilization—an interval that presumably normalizes energy balance. Consequently, while tailored interventions addressing diminished energy expenditure remain crucial for long-term success, these newer findings point to the possibility that AT might be less pronounced than once assumed, emphasizing the need for additional high-quality research to ascertain its real-world impact on long-term weight management.

## 5. Immune System, Meta-Inflammation, and Obesity

Obesity is increasingly recognized as a state of chronic low-grade inflammation, often termed “meta-inflammation” (metabolically driven inflammation), which arises from complex interactions between the immune system, adipose tissue, and metabolic organs. This persistent immune activation contributes to insulin resistance, dyslipidemia, and an increased risk of cardiovascular/renal/hepatic/metabolic diseases [45].

Adipose tissue plays a central role in obesity-induced immune dysregulation. As adipocytes expand, they undergo stress and apoptosis, triggering the infiltration of immune cells, particularly pro-inflammatory M1 macrophages, which secrete cytokines such as tumor necrosis factor-alpha (TNF-α), interleukin-6 (IL-6), and interleukin-1β (IL-1β). These cytokines disrupt insulin signaling, promote oxidative stress, and perpetuate systemic inflammation. Conversely, in lean individuals, adipose tissue is predominantly populated by anti-inflammatory M2 macrophages and regulatory T cells (Tregs), which help maintain metabolic homeostasis [45].

Beyond cytokines, adipokines, the bioactive molecules secreted by adipose tissue, play a crucial role in the systemic meta-inflammatory response in obesity. In obesity, the balance between pro-inflammatory and anti-inflammatory adipokines is disrupted, exacerbating metabolic dysfunction. Leptin, which regulates appetite and energy homeostasis, is elevated in obesity but paradoxically fails to exert its anorexigenic effects due to leptin resistance. Conversely, adiponectin, an anti-inflammatory and insulin-sensitizing adipokine, is significantly reduced in obesity, further contributing to insulin resistance and chronic inflammation. Other dysregulated adipokines, such as resistin, visfatin, and chemerin, promote endothelial dysfunction, increase oxidative stress, and enhance inflammatory signaling via Nuclear Factor Kappa-Light-Chain Enhancer of Activated B Cells (NF-κB) and Janus Kinase-Signal Transducer and Activator of Transcription (JAK-STAT) pathways. These systemic alterations reinforce the meta-inflammatory state and drive obesity-related cardiovascular/renal/hepatic/metabolic diseases [6].

The gut microbiota is increasingly recognized as a key contributor to obesity-induced meta-inflammation. In individuals with obesity, the gut microbiome exhibits dysbiosis, characterized by a higher Firmicutes-to-Bacteroidetes ratio, reduced microbial diversity, and an increase in pro-inflammatory pathobionts. This altered microbiome composition promotes gut permeability, allowing bacterial endotoxins such as lipopolysaccharides (LPSs) to enter the circulation, a phenomenon known as metabolic endotoxemia. LPSs activate toll-like receptor 4 (TLR4) signaling on immune cells, leading to NF-κB activation, increased cytokine production, and systemic inflammation. Additionally, dysbiotic microbiota produce short-chain fatty acids (SCFAs) that influence immune function, with alterations in butyrate levels contributing to increased inflammation and metabolic dysfunction. Restoring gut microbial balance through prebiotics, probiotics, and dietary interventions has been proposed as a potential strategy to mitigate meta-inflammation in obesity [46].

Hypothalamic inflammation plays a critical role in the dysregulation of energy balance and metabolic homeostasis during obesity. The hypothalamus, particularly the arcuate nucleus, is responsible for integrating hormonal and neuronal signals that regulate appetite and energy expenditure. In obesity, inflammatory signaling within the hypothalamus is heightened, driven by microglial activation and an increased expression of TNF-α, IL-6, and IL-1β. This inflammation impairs the function of pro-opiomelanocortin (POMC) and agouti-related peptide (AgRP) neurons, leading to leptin and insulin resistance in the central nervous system. As a result, satiety signals are blunted, hunger is increased, and energy expenditure is reduced, perpetuating weight gain and metabolic dysfunction. Targeting hypothalamic inflammation through anti-inflammatory agents, lifestyle modifications, and neuromodulatory therapies represents a promising avenue for obesity treatment [47].

Obesity is paradoxically associated with both chronic inflammation and immune suppression, making individuals with obesity more susceptible to infections and impaired immune responses. Excess adiposity alters T cell functions, reducing naïve T cell populations and increasing senescent and exhausted T cells, which impairs adaptive immunity. Obesity is also associated with dysregulated B cell responses, leading to reduced antibody production and weaker responses to infections and vaccinations. Furthermore, elevated levels of IL-6 and TNF-α interfere with the normal function of natural killer (NK) cells and macrophages, diminishing their ability to clear pathogens. The chronic meta-inflammatory state in obesity also disrupts hematopoiesis, leading to impaired neutrophil function and reduced antigen-presenting cell activity. Clinically, this immune dysfunction translates into an increased risk of severe viral infections (e.g., influenza, COVID-19), delayed wound healing, and poor vaccine efficacy in individuals with obesity [48,49]. 

## 6. Clinical Implications

Building upon the evidence that metabolic drivers extend well beyond standard calorie accounting, clinical strategies aimed at effective weight management need to integrate multiple elements beyond simply “eat less, move more”. Below are key recommendations and open questions to guide both clinical practice and future investigations:

▪ Individualized Metabolic Assessments
  ○ Indirect Calorimetry in Specific Cases: Patients who struggle with weight management despite diligent adherence may benefit from direct measurements of RMR and substrate oxidation to identify any underestimation or overestimation of caloric needs.
  ○ Gut Microbiome Profiling and Nutrient Absorption Metrics: Given emerging data linking the gut microbiota to nutrient extraction, advanced screening (e.g., metagenomic or metabolomic approaches) could pinpoint maladaptive microbial compositions that potentiate excessive calorie harvest.
▪ Emphasis on High-Protein and High-Fiber Foods and Meal Timing:
  ○ Since DIT is consistently higher for protein and slowly digested carbohydrate sources, focusing on high-quality, whole food meals may yield a more substantial thermogenic response.
  ○ Meal Timing and Composition Adjustments: Late-night eating appears to lower energy expenditure and elevate fat storage signals, suggesting that clinicians might counsel patients to align larger meals with earlier circadian phases, pending individual tolerances and lifestyle constraints.
▪ Physical Activity and NEAT Strategies Mitigating Compensatory Behaviors: Structured exercise protocols should include guidance on NEAT throughout the day (e.g., standing breaks, walking during calls). Monitoring step counts or fidgeting can help reduce unintentional drops in overall daily energy expenditure.
▪ BAT Stimulation: While not a stand-alone solution, mild cold exposures or the inclusion of thermogenic dietary factors (capsaicin and catechins) may modestly augment total daily energy output, particularly in individuals with detectable BAT.
▪ AT and Weight Stabilization
  ○ Weight-Stabilization Intervals: Emerging evidence indicates that AT may be transient or attenuated after a period of neutral energy balance. Clinicians could incorporate structured “maintenance phases” into weight-loss programs to allow metabolic rates to recalibrate before pushing for further fat loss.
  ○ “Cheat Meals or Days”: Incorporating meals or days of increased energy intake in the form of whole foods might be beneficial for some individuals in preventing AT.
  ○ Standardized Testing for AT: Reliable clinical protocols (e.g., repeated metabolic assessments under varied caloric intakes) might help identify patients particularly prone to AT so that interventions (e.g., thermogenic aids or refeed strategies) can be initiated proactively.

## 7. Limitations, Challenges, and Future Research Directions

While substantial progress has been made in elucidating the complex factors governing the human energy balance, significant limitations persist in both research methodologies and the clinical translation of findings. Addressing these gaps is essential to refining obesity management strategies and identifying more precise interventions for weight control. 

### 7.1. Limitations of Current Research

▪ Heterogeneity in Study Populations: Many studies investigating metabolic adaptation, thermogenesis, and fecal/urinary energy losses are conducted on small or highly specific populations (e.g., athletes, individuals with obesity, or those with metabolic disorders), limiting the generalizability of findings to broader populations.
▪ Short-Term vs. Longitudinal Data: Most energy balance studies rely on short-term calorimetry or feeding trials, providing only snapshots of metabolic responses. Longitudinal studies are needed to assess how adaptive changes in metabolism, gut microbiota, and thermogenesis influence long-term weight trajectories.
▪ Interindividual Variability in Energy Expenditure: Standard metabolic equations fail to account for substantial interindividual variability in BMR, AT, and NEAT. Direct measurement via indirect calorimetry is still not widely implemented in routine clinical practice due to cost and accessibility.
▪ Lack of Standardized Protocols for AT: Research on AT remains inconsistent, with some studies showing substantial declines in the metabolic rate following weight loss, while others suggest AT is transient. Standardized, controlled trials that incorporate multiple weight-stabilization checkpoints are needed to determine the true impact of AT on long-term weight management.
▪ Gut Microbiota and Energy Balance: Although emerging evidence links gut microbiota composition to energy extraction and systemic inflammation, the exact mechanisms remain unclear. Most studies rely on 16S rRNA sequencing, which lacks the resolution to fully characterize microbial metabolic function. More robust metagenomic and metabolomic approaches are needed.
▪ Underestimation of Behavioral and Environmental Influences: While metabolic factors are critical, research often underestimates the impact of psychological, behavioral, and socioeconomic influences on obesity. Stress, sleep deprivation, food availability, and urbanization all modulate energy intake and expenditure and should be integrated into comprehensive models.

### 7.2. Future Research Imperatives

To bridge these knowledge gaps, future studies should focus on integrating precision metabolic assessments, digital health tools, and AI-driven analytics to optimize obesity interventions.

▪ Longitudinal Gut–Metabolism Studies: Prospective studies that integrate microbiome sequencing with precise fecal energy loss measurements can clarify how microbial communities influence nutrient absorption, thermogenesis, and fat storage.
▪ Cold Exposure and BAT Activation Trials: Larger, controlled investigations on the effects of daily cold exposure, pharmaceutical BAT activators, and dietary thermogenic agents (e.g., capsinoids, catechins) can provide deeper insights into sustainable metabolic interventions.
▪ AT Research: Randomized trials incorporating repeated metabolic assessments across different caloric intakes and weight-maintenance phases can help quantify the persistence and clinical relevance of AT.
▪ Digital Health and AI-Driven Metabolic Monitoring: Implementation studies on real-time metabolic tracking (e.g., continuous glucose monitors, wearable indirect calorimeters) can assess the effectiveness of personalized weight management interventions. AI-powered algorithms could help predict individual responses to specific diets, exercise regimens, and thermogenic strategies.
▪ Intervention Studies on Meal Timing and Circadian Biology: Given the growing evidence that circadian misalignment affects metabolism, well-designed trials on time-restricted feeding, chrononutrition, and metabolic rate fluctuations throughout the day are necessary.
▪ Multi-Omics Approaches to Personalized Obesity Treatment: The integration of genomics, transcriptomics, metabolomics, and gut microbiome profiling could redefine metabolic phenotypes and tailor interventions beyond the standard calorie-deficit model.

By addressing these limitations and pursuing these research directions, the field of human energy balance and obesity management can move toward more individualized, precision-based interventions that improve metabolic health and long-term weight outcomes.

## 8. Conclusions

This manuscript underscores that human energy balance is not a simple ledger of “calories in versus calories out”. Weight trajectories are shaped by a diverse array of processes, from basal metabolic rate variability and DIT to BAT function, NEAT, and fecal/urinary nutrient excretion. These factors can substantially deviate real-world outcomes from those predicted by conventional calorie-based formulas. Contemporary findings reveal how individuals differ widely in their susceptibility to weight gain or weight loss, due to interwoven mechanisms such as thrifty or spendthrifty phenotypes, the dynamic nature of AT, and the extent of unabsorbed dietary energy. Given this complexity, clinicians must integrate multiple dimensions, ranging from direct RMR measurements to careful macronutrient and timing strategies, as well as assessing gut microbiota profiles, to craft interventions that align more closely with each patient’s metabolic identity. Ultimately, the rise of precision nutrition and digital health platforms offers a path toward genuinely personalized regimens, where real-time metabolic feedback guides incremental adjustments in diet, activity, and behavior. Embracing these advanced, multifactorial insights may finally move obesity care beyond the limitations of static calorie-based prescriptions, delivering more effective and enduring solutions for weight control.

## Figures and Tables

**Table 1:** Additional Factors affecting diet-induced thermogenesis. Abbreviations: basal metabolic rate, BMR; body mass index, BMI; diet-induced thermogenesis, DIT; fat-free mass, FFM; kilocalories, kcal; kilojoules, kJ; long-chain triglycerides, LCT; medium-chain triglycerides, MCT; probability value, *p*-value; resting metabolic rate, RMR; thermic effect of food, TEF.

Study	Design	Findings	Implications
[23]	n = 277 (136 older adults aged 60–88 years and 141 younger adults aged 18–35 years); indirect calorimetry; 4 h post-meal assessment	Older adults had lower BMR (*p* = 0.01) and TEF (6.4% vs. 7.3%, *p* = 0.02), reducing daily energy expenditure by ~65 kcal/day; Postprandial insulin levels higher in older adults (8072 vs. 4476 pmol/4 h; *p* < 0.0001).	Age-related declines in BMR and TEF may predispose older adults to weight gain, requiring adjustments in dietary intake and physical activity.
[24]	36 men (active vs. sedentary); cross-sectional comparison	TEF was 45% higher in active younger men (323.42 kJ vs. 222.17 kJ, *p* < 0.01) and 31% higher in active older men (292.04 kJ vs. 215.47 kJ, *p* < 0.01).	Habitual physical activity is associated with greater postprandial energy expenditure, reinforcing its role in weight management.
[25]	Meta-analysis of 19 studies (54 treatment arms) on DIT; subgroup analysis on MCT vs. LCT; further analysis on meal size effect.	- For every 100 kJ increase in meal energy intake, DIT increased by 1.1 kJ/h (*p* < 0.001); adjusted for age, BMI, sex, duration: 1.2 kJ/h (*p* < 0.001). - MCT vs. LCT (3 studies): MCT significantly increased DIT (*p* = 0.002), with MCT meals yielding up to 29.4 kJ/h vs. 21.9 kJ/h for LCT. - Single-bolus vs. multiple small meals: One large meal generated a significantly higher DIT (*p* = 0.02), with differences ranging from 10 to 15 kJ/h.	- Higher energy intake leads to proportional increases in DIT, supporting the role of meal size in postprandial thermogenesis. - MCTs are more thermogenic than LCTs, suggesting a dietary strategy for increasing energy expenditure. - Consuming a single large meal may enhance DIT more than spreading the same energy intake across multiple meals.
[26]	Crossover study, 17 healthy participants, comparing isoenergetic meals of whole vs. processed foods (multi-grain bread and cheddar cheese vs. white bread and processed cheese product)	Whole food meals elicited a significantly higher thermic effect (19.9% ± 2.5% of meal energy) vs. processed food meal (10.7% ± 1.7%), *p* = 0.005. This corresponded to a 46.8% greater total diet-induced thermogenesis (DIT) (*p* = 0.0009). Processed food meals led to approximately 9.7% more net assimilated energy. Whole food meal was rated more palatable (*p* = 0.005), but no significant difference in satiety ratings between meal types.	- Whole food meals significantly enhance postprandial energy expenditure, supporting their role in weight management. - Processed foods may lead to higher net energy retention, contributing to positive energy balance and obesity risk. - Dietary recommendations should prioritize minimally processed foods to maximize energy expenditure and improve metabolic health.
[27]	9 participants; small controlled trial measuring TEF at different times of day	TEF was significantly higher in the morning than in the afternoon (*p* = 0.02) and trended higher in the afternoon than in the evening (*p* = 0.06).	Aligning meals with morning metabolism may optimize TEF and energy balance.
[28]	14 overweight/obese individuals; TEF measured across breakfast, lunch, and dinner	- TEF in the morning (60.8 kcal ± 5.6) was 1.6× higher than at lunch (36.3 kcal ± 8.4) and 2.4× higher than at dinner (25.2 kcal ± 9.6), *p* = 0.022. - When TEF was adjusted for circadian fluctuations in RMR using a sinusoidal model, the differences were nullified: breakfast (54.1 kcal ± 30.8), lunch (49.5 kcal ± 29.4), dinner (49.1 kcal ± 25.7), *p* = 0.680.	TEF variability is largely explained by circadian rhythm rather than meal timing, suggesting meal composition may be a more critical factor.

## Data Availability

Not applicable.

## Abbreviations

The following abbreviations are used in this manuscript:

18F-FDG PET	Fluorodeoxyglucose Positron Emission Tomography
AACS	Acetoacetyl-CoA Synthetase
ABHD5	Abhydrolase Domain Containing 5
ACL	ATP Citrate Lyase
ASAH1	N-Acylsphingosine Amidohydrolase 1
AT	Adaptive Thermogenesis
ATP	Adenosine Triphosphate
BMR	Basal Metabolic Rate
BAT	Brown Adipose Tissue
CKM	Cardiovascular–Kidney–Metabolic
CRHM	Cardiovascular–Renal–Hepatic–Metabolic
DECR1	2,4-Dienoyl-CoA Reductase 1
DIT	Diet-Induced Thermogenesis
EAT	Exercise Activity Thermogenesis
EE	Energy Expenditure
EPOC	Excess Post-Exercise Oxygen Consumption
FFM	Fat-Free Mass
GPAQ	Global Physical Activity Questionnaire
GPAM	Glycerol-3-Phosphate Acyltransferase, Mitochondrial
IPAQ	International Physical Activity Questionnaire
LCT	Long-Chain Triglycerides
MAPK	Mitogen-Activated Protein Kinase
MCT	Medium-Chain Triglycerides
MICE	Moderate-Intensity Continuous Exercise
NEAT	Non-Exercise Activity Thermogenesis
PAR	Physical Activity Ratio
PEE	Physical Activity-Related Energy Expenditure
PLD6	Phospholipase D Family Member 6
RMR	Resting Metabolic Rate
SIE	Sprint Interval Exercise
TEF	Thermic Effect of Food
TGF-β	Transforming Growth Factor Beta
UCP1	Uncoupling Protein 1
VO2 max	Maximal Oxygen Consumption
