# Correction: Theodorakis, N.; Nikolaou, M. The Human Energy Balance: Uncovering the Hidden Variables of Obesity. *Diseases* 2025, *13*, 55

**DOI:** 10.3390/diseases13040108

**Published:** 2025-04-03

**Authors:** Nikolaos Theodorakis, Maria Nikolaou

**Affiliations:** 1 NT-CardioMetabolics, Clinic for Metabolism and Athletic Performance, 47 Tirteou Str., 17564 Palaio Faliro, Greece; 2 Department of Cardiology & Preventive Cardiology Outpatient Clinic, Amalia Fleming General Hospital, 14, 25th Martiou Str., 15127 Melissia, Greece; m.nikolaou@flemig-hospital.gr; 3 School of Medicine, National and Kapodistrian University of Athens, 75 Mikras Asias, 11527 Athens, Greece

## Text Correction

There was an error in the original publication [1]. The third paragraph in Section 3.6 was mistakenly retained after an improved version was incorporated into the second paragraph, resulting in redundancy. Additionally, references 38 and 39 should be included in the second paragraph.

A correction has been made to Section 3.6, Paragraphs 2 and 3. The revised version of Section 3.6 is as follows:

“EAT refers to the energy spent during structured physical activities, ranging from moderate-intensity continuous exercise (MICE) to high-intensity interval exercise (HIIE) and sprint interval exercise (SIE), as well as resistance training. Although exercise typically accounts for 0–10% of the total daily energy output, these activities can acutely raise overall energy expenditure—sometimes substantially—yet compensatory behaviors may diminish the net benefits. Individuals might unintentionally reduce non-exercise activity after intense workouts, or increase caloric intake in response to heightened appetite. Consequently, exercise regimens designed to monitor and mitigate these compensations often produce more consistent outcomes for weight management [2,37].

Beyond the immediate calories burned, exercise can also affect energy metabolism through excess post-exercise oxygen consumption (EPOC). A recent systematic review of 22 studies examined the effect of exercise intensity—HIIE vs. MICE vs. SIE—on EPOC, splitting investigations into those evaluating short-duration EPOC (≤3 h) and long-duration EPOC (>3 h). Among short-duration evaluations that subtracted baseline energy expenditure (EE), HIIE produced ~136 kJ of post-exercise EE, while MICE averaged ~101 kJ. SIE reached ~241 kJ, compared with ~151 kJ for MICE. In long-duration measurements, HIIE resulted in ~289 kJ, whereas MICE averaged ~159 kJ; no long-duration data were available for SIE vs. MICE comparisons. These findings suggest that EE from EPOC tends to be greater following HIIE and SIE than with MICE, and that longer measurement intervals may reveal higher EPOC totals. More standardized methodologies remain necessary to delineate the effective duration of EPOC after such training protocols [38,39].

In one investigation, participants who engaged in 80 min of endurance exercise at approximately 70% of their maximal oxygen uptake exhibited sustained elevations in oxygen uptake for up to 12 h, with potential metabolic effects persisting for as long as 24 h. Notably, post-meal oxygen consumption was also elevated following exercise, indicating that endurance training may enhance metabolic efficiency even during subsequent nutrient intake. These findings reinforce the importance of both exercise intensity and duration in modulating post-exercise metabolic responses, suggesting that structured training strategies can contribute to an improved energy balance and long-term metabolic health [40].”

The authors state that the scientific conclusions are unaffected. This correction was approved by the Academic Editor. The original publication has also been updated.
